# Non‐invasive characterization of melanoma depth at single‐cell resolution

**DOI:** 10.1111/jdv.20811

**Published:** 2025-07-03

**Authors:** Juan Aguirre, Christine Gasteiger, Benedikt Hindelang, Markus Seeger, Andrei Bereznhoi, Ina Weidenfeld, Ulf Darsow, Andre C. Stiel, Susanne Annette Steimle‐Grauer, Christian Posch, Tilo Biedermann, Vasilis Ntziachristos

**Affiliations:** ^1^ Chair of Biological Imaging, Central Institute for Translational Cancer Research (TranslaTUM), School of Medicine and Health & School of Computation, Information and Technology Technical University of Munich Munich Germany; ^2^ Institute of Biological and Medical Imaging, Bioengineering Center Helmholtz Zentrum München Neuherberg Germany; ^3^ Departamento de Tecnología Electrónica y de las Comunicaciones Universidad Autonoma de Madrid Madrid Spain; ^4^ Instituto de Investigacion Sanitaria de la Fundacion Jimenez Diaz Madrid Spain; ^5^ Department of Dermatology and Allergology Technical University of Munich Munich Germany; ^6^ Protein Engineering for Superresolution Microscopy Lab University of Regensburg Regensburg Bavaria Germany; ^7^ Department for Dermatology Vienna Healthcare Group, Clinic Hietzing Vienna Austria; ^8^ Sigmund Freud University Vienna Faculty of Medicine Vienna Austria; ^9^ Munich Institute of Robotics and Machine Intelligence (MIRMI) Technical University of Munich Munich Germany

**Keywords:** cells, imaging, melanoma, melanoma classification

## Abstract

**Background:**

Efficient staging and management of cutaneous melanoma, one of today's deadliest skin cancers, requires non‐invasive determination of tumour depth (Breslow depth). However, current imaging technologies lack the necessary contrast or penetration to measure Breslow depth.

**Objectives:**

To determine if raster‐scanning optoacoustic mesoscopy (RSOM) can fill this gap in dermatology.

**Methods:**

We used phantoms to optimize RSOM for melanoma imaging and demonstrated its capability to image melanocytes at single‐cell resolution in deep tissue. We then compared RSOM's ability to measure Breslow depth against the clinical standard in a pilot study.

For the phantoms studies, we compared the ability of an optimized RSOM system to measure the concentration and diameter of single melanoma cells against gold‐standard microscopy methods. For the pilot clinical study, we used linear regression to compare RSOM's Breslow depth against the clinical standard, obtaining the goodness of fit (*R* ^2^) and the *p*‐value.

For the pilot clinical study, we imaged nine lesions: 7 superficially spreading melanomas, 1 benign dysplastic nevus and 1 blue nevus. The average age of the patients was 56.2 ± 12.5 years. We also imaged 10 non‐lesional skin areas from healthy volunteers.

**Results:**

By utilizing ultra‐wideband frequency detection and optimized illumination wavelength, we show that RSOM achieves non‐invasive imaging of melanoma at the resolution of single melanocytes, penetrating more than 3 mm into the skin. The agreement between RSOM and the standard‐of‐care histological assessment was *R* ^2^ = 0.886 (*p* = 0.0002) for Breslow depth determination.

**Conclusions:**

RSOM provides non‐invasive imaging performance that correlates with the Breslow depth determination. Further work is needed to confirm these findings and to test RSOM against other non‐invasive methods.

## Graphical abstract

Non‐invasive determination of Breslow depth would be beneficial for the clinical management of melanoma. We demonstrate that RSOM can achieve non‐invasive imaging of single melanocytes up to several millimetres in tissue. A pilot clinical study showed a high degree of correlation between RSOM and the standard for Breslow depth determination.

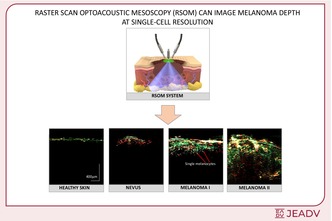

**Why was the study undertaken?**

Non‐invasive imaging methods are demanded for Breslow depth determination. We investigated the performance of RSOM for non‐invasive imaging of melanoma.

**What does this study add?**

We demonstrate that optimized RSOM can achieve non‐invasive imaging of single melanocytes up to several millimetres deep in tissue. A pilot clinical study showed a positive correlation between RSOM and the clinical standard for Breslow depth determination.

**What are the implications of this study for disease understanding and/or clinical care?**

The ability of imaging melanoma cells non‐invasively at single‐cell resolution may fill this gap in dermatology, with several implications for the management and research of melanoma. Further work is needed to confirm these findings and to test RSOM against other non‐invasive methods.

## INTRODUCTION

Cutaneous melanoma is a leading cause of death among the young population.^1^ The American Joint Commission on Cancer (AJCC) 2018 guidelines state that the primary lesion's depth (Breslow depth) and tumour ulceration are crucial for staging.^2^ A Breslow depth under 1 mm indicates low metastasis risk,^2^ while deeper lesions require larger excision margins and possibly sentinel lymph node biopsy.^3^ Today, Breslow depth determination requires biopsies that can disrupt lymphatic drainage and the tumour microenvironment,^4^, ^5^, ^6^, ^7^ the latter not being optimal in times of upcoming neoadjuvant treatment strategies. Moreover, non‐invasive Breslow depth assessment could improve surgical planning, reducing the number of biopsies by allowing precise excisions for tumours over 1 mm.^8^

In current practice, Breslow thickness is measured using histological 2D slices following AJCC guidelines and others.^2^, ^3^ This method identifies the maximum depth of melanoma cells from the stratum granulosum at single‐cell resolution. Non‐invasive methods for measuring Breslow thickness should meet the AJCC standards.^9^, ^10^ Optical methods do not yield penetration depths appropriate for a comprehensive tumour thickness assessment.^11^, ^12^ Reflectance confocal microscopy (RCM) cannot penetrate deeper than 200 μm due to light scattering,^13^ while optical coherence tomography (OCT) can only image up to 1 mm deep and lacks the cellular contrast needed to detect single melanoma cells.^11^, ^12^, ^14^ Multispectral diffuse optical methods can probe deeper than RCM and OCT, achieving, in the best‐case scenario, a resolution equal to one third of the imaging depth. Therefore, their performance is not adequate for Breslow depth determination.^15^

Melanoma ultrasonography at frequencies ranging from 7.5 to 100 MHz^7^, ^8^, ^15^, ^16^, ^17^, ^18^, ^19^, ^20^, ^21^, ^22^, ^23^, ^24^, ^25^ enables imaging depths of several millimetres but lacks contrast for detecting melanocytes.^17^, ^21^, ^22^, ^23^, ^24^ On the other hand, optoacoustic (photoacoustic) methods have the potential to offer similar imaging performance to ultrasonography, but with higher contrast for detecting melanocytes, due to the high signals offered by melanin's absorption of light.^16^, ^19^, ^20^ However, in its standard implementation (central frequencies of ~25 MHz), optoacoustic imaging has failed to meet AJCC recommendations for Breslow thickness, yielding resolutions in the >100 μm range.

In this study, we interrogated whether Raster Scanning Optoacoustic Mesoscopy (RSOM) could meet the AJCC recommendations. We utilized ultra‐wide bandwidth (UWB‐RSOM) detection (~10–110 MHz)^26^, ^27^ for a 10 μm axial resolution and investigated the wavelength that could offer an optimal balance between depth and contrast. We used the system to image healthy skin and various melanocytic lesions in patients, estimating the Breslow and validating the results against histology. We demonstrate, for the first time, non‐invasive tumour thickness determination at single‐cell resolution, which can be combined with tumour microvascular imaging.^28^ Finally, we discuss the potential of UWB‐RSOM to enhance melanoma research and healthcare.

## RESULTS

**FIGURE 1:**
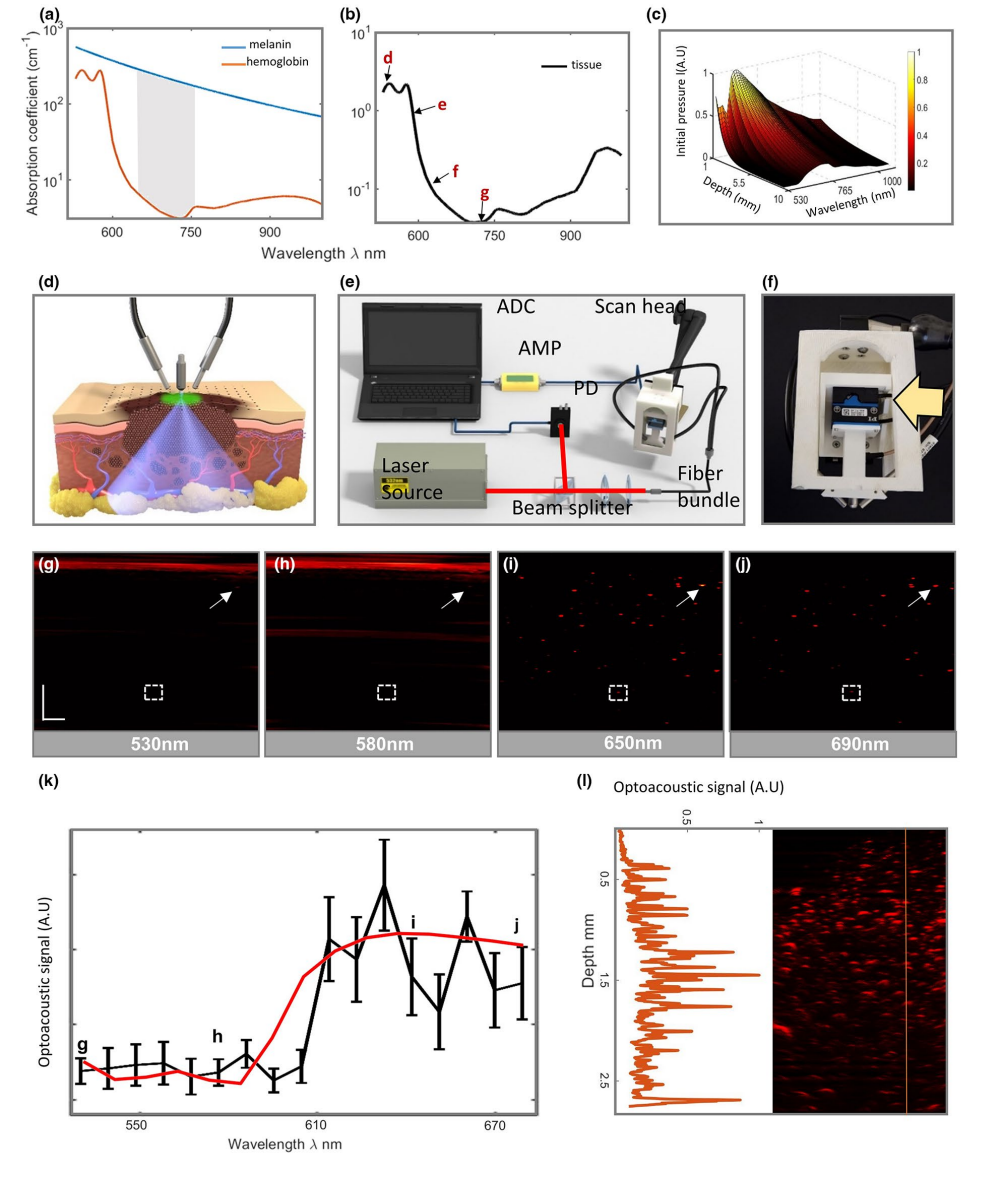
Contrast to noise ratio (CNR) and signal to noise ratio (SNR) optimization. (a) Absorption coefficient spectra of melanin and 60% oxygenated haemoglobin. The grey area indicates the region in which the difference between melanin and haemoglobin absorption is at its maximum. (b) Absorption coefficient of the dermis assuming a composition of 10% blood (60% oxygenation) and 70% water. The spectral points corresponding to figures d–g are marked with black arrows. (c) Initial pressure as function of depth and wavelength, calculated theoretically for skin comprising 10% blood (60% oxygenation) and 70% water. (d) Experimental raster‐scanning optoacoustic mesoscopy (RSOM) setup employed for melanocyte detection. The focal point of the transducer is located above the skin. Two fibre bundles deliver the illumination energy. The transducer and the bundles are raster‐scanned over a rectangle. For each point, one optoacoustic wave is generated and recorded. (e) Scheme of the optimized ultra‐wide bandwidth RSOM (UWB‐RSOM). A light pulse is generated from the optical parametric oscillator (OPO) laser head, and the pulse is divided by a beam splitter. Part of the beam triggers data acquisition for analogue to digital conversion (ADC). The other part of the beam is coupled to a fibre bundle. The fibre bundle directs the illumination light onto the skin surface. ADC, analogue to digital convertor; AMP, amplifier; CL, concave lens; FL, filter; PBS, beam splitter; PD, photodiode; UT, ultrasound transducer. (f) Side view of the mesoscope head. The Z stage can be seen (arrow), which allows for fine adjustment of the height of the transducer with respect to the surface of the skin. (g–j) Reconstructed images of a biological phantom containing water, blood, lipid and 400 melanoma cells/μL acquired at 530 nm (g), 580 nm (h), 650 nm (i) and 690 nm (j). The white arrows point to a cell close to the surface that is visible in all four images. The white dashed box represents the region of interest in which the spectrum shown in (k) is calculated. (k) Experimental optoacoustic spectrum of cells (black line) situated 1.8 mm deep in the images shown in (g–j) together with its theoretical counterpart taken from (c). (l) Profile taken in the depth direction corresponding to the line shown in the adjacent image. The image corresponds to the maximum intensity projection of a biological phantom containing water, blood and intralipid and a concentration of 400 melanoma cells/μL. Scale bars: 400 μm.

We first aimed to identify optimal wavelengths for penetrating several millimetres into the skin while maximizing melanoma cell contrast. Achieving a balance between signal generation and light attenuation in tissue is critical for differentiating melanin and haemoglobin. In the visible range (300–600 nm), melanin's absorption is high, producing a strong optoacoustic signal. However, haemoglobin also absorbs strongly, limiting penetration depth to 1–1.5 mm and reducing melanin contrast (Figure 1a,b). In the near‐infrared (NIR) range (600–900 nm), melanin absorption decreases, lowering the optoacoustic signal, but haemoglobin absorption drops steeply above 610 nm (Figure 1b), allowing deeper light penetration and better melanin contrast. We computed optoacoustic pressure for different wavelengths, using a tissue model of melanosomes in dermis with 10% blood and 90% water. Wavelengths in the far‐red range (620–690 nm) (Figure 1c) provided the best balance between contrast and depth, maximizing contrast‐to‐background ratio (CNR) and melanin signals (see Appendix S1).

Having identified the optimal CNR region with simulations, we aimed to determine if melanocytes could produce sufficient optoacoustic signals in the far‐red region for optoacoustic mesoscopy, and to experimentally confirm these simulations. We created a dermis‐simulating phantom with 10% blood, 10% intralipid, and 80% water, containing 400 melanoma cells per μL (see Methods). Using an RSOM system with an ultra‐wideband spherically focused ultrasound detector, we scanned the phantom to collect optoacoustic signals at 10–120 MHz.^26^, ^29^, ^30^ Different wavelengths for illumination were achieved with an optical fibre connected to a tunable OPO laser covering 400–1000 nm (see Figure 1d–f and Methods). The scan‐head, containing the detector and fibre illuminator, was mounted on an articulated arm for flexible positioning (Figure 1f). Laser pulse energy and repetition rate adhered to human‐use safety limits as per the American National Standard for Safe Use of Lasers.

Imaging at the visible region (530 nm and 580 nm) confirmed that contrast in the visible region is low due to strong haemoglobin absorption (Figure 1c). Only a few cells near the surface of the phantom (maximum depth of ~300 μm) (Figure 1g,h), can be observed together with strong signals from superficial blood which also led to reflection artefacts (Figure 1h). However, in the 620–690 nm range, melanoma cell signals were visible throughout the 2 mm depth of the phantom (Figure 1i,j). The optoacoustic spectrum of a melanoma cell at 1.8 mm depth (Figure 1k) confirmed that the 620–690 nm range is optimal for visualizing single melanoma cells beyond 1 mm depth (Figure 1c). At 650 nm, melanoma cells were visible at depths of at least 3 mm (Figure 1l).

### Bandwidth and single melanocyte detection

**FIGURE 2:**
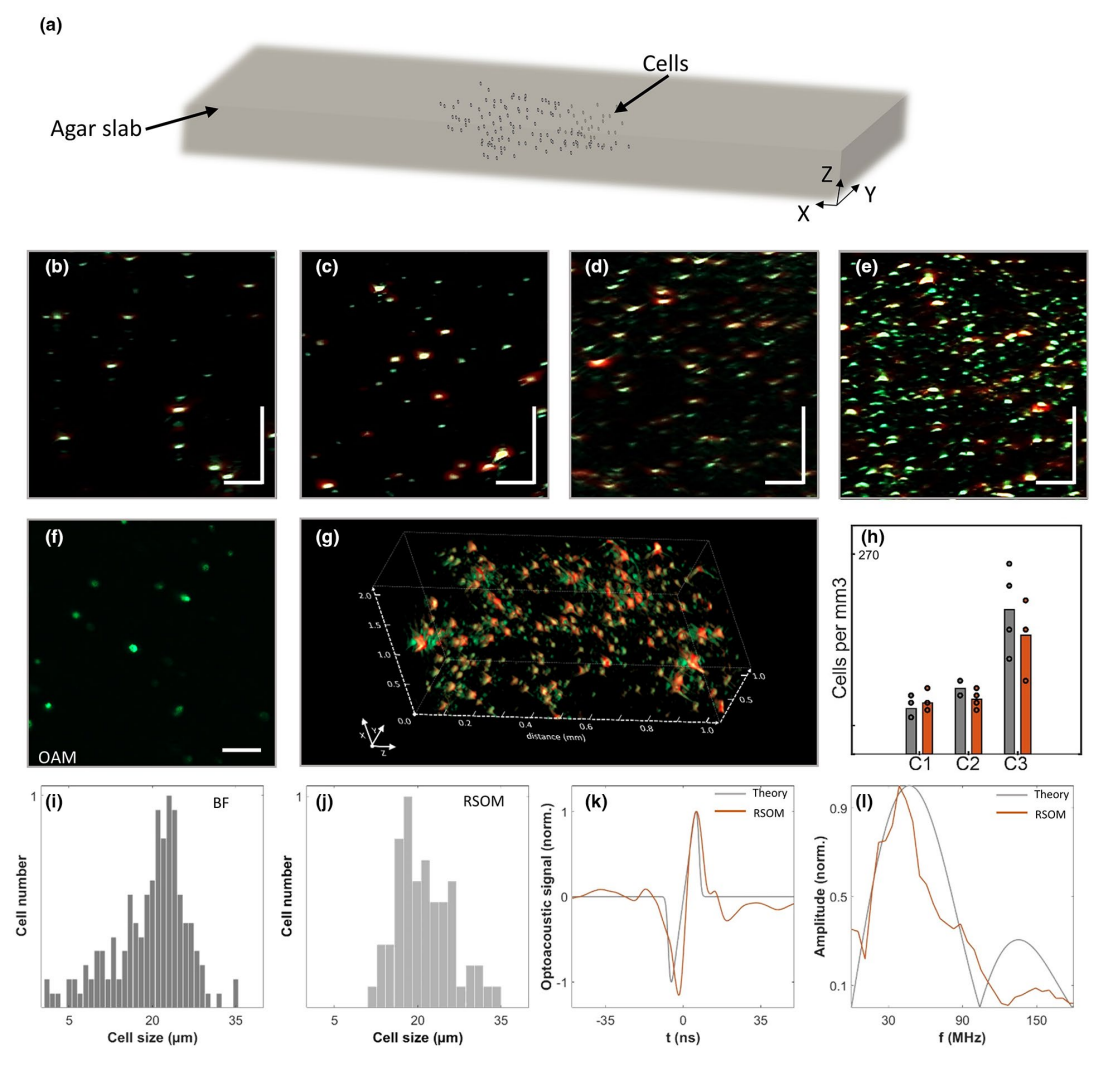
Ultra‐wide bandwidth raster‐scanning optoacoustic mesoscopy (UWB‐RSOM) can image individual melanoma cells, as shown in this acoustic study. (a) Scheme of the phantoms used. Four agar slabs, each containing an increasing number of B16F10 cells, were prepared to test the single‐cell imaging ability of UWB‐RSOM. (b–e) UWB‐RSOM cross‐sectional view of each phantom, ordered by increasing number of melanoma cells. Scale bar: 100 μm. (f) Optoacoustic microscopy (OAM) image of phantom 2. Scale bar: 100 μm. (g) Render of the 3D UWB‐RSOM image of phantom 2. (h) Concentration of melanoma cells as measured by OAM and UWB‐RSOM. (i, j) Histograms of the sizes of melanoma cells obtained by bright‐field microscopy (i) and UWB‐RSOM (j). (k) Simulated optoacoustic signal of a melanocyte (20 μm diameter spherical cell) and the optoacoustic signal generated by a real melanocyte captured by UWB‐RSOM. (l) Frequency content of the signals shown in (k).

To characterize the single cell imaging abilities of UWB‐RSOM, we constructed four transparent agar cubes (Phantoms 1–4) with increasing concentrations of B16F10 melanoma cells (see Methods). Agar was used to mimic tissue acoustic properties (Figure 2a). The cells were confined in a 3D volume of ~4 × 4 × 2 mm^3^ (*x*, *y*, *z*) and scanned using a 4 × 4 mm grid over >2 mm depth. The phantoms were made optically transparent to allow for high‐resolution optical imaging observations.

Imaging was performed with UWB‐RSOM and optoacoustic microscopy (OAM) at 650 nm, the latter used as a reference (Appendix S1). UWB‐RSOM maximum amplitude projection (MIP) images of each phantom (Figure 2b–e) showed point‐like structures increasing in density with higher melanocyte concentration. These points are colour‐coded: red for lower frequencies (larger structures) and green for higher frequencies (smaller structures). OAM scans confirmed individual melanoma cells in phantoms 1–3 and some aggregation in phantom 4 (Video [Link], [Link], [Link], [Link]), aligning with UWB‐RSOM images (Figure 2b–e). The OAM MIP of phantom 2 (Figure 2f) displayed cells matching in distribution, size and concentration to the UWB‐RSOM image (Figure 2c). This agreement extends to fly‐through videos in the z direction (Video [Link], [Link], [Link], [Link], [Link], [Link], [Link], [Link]), despite OAM having better resolution (10 μm vs. ~30 μm lateral). A UWB‐RSOM rendering of phantom 2 is shown in Figure 2g. Quantitative comparisons of cell concentrations from OAM and UWB‐RSOM images showed 90%, 83% and 82% agreement in phantoms 1–3, respectively (Figure 2h, see methods for details).

We quantitatively assessed UWB‐RSOM's ability to resolve single melanocytes by comparing cell diameters from phantom 2 obtained via bright field cytometry and UWB‐RSOM (see Methods). Histograms from both methods show similar trends (Figure 2i,j). Bright field microscopy yielded a mean diameter of 21.3 μm, compared to 19.7 μm from UWB‐RSOM, indicating a 93% agreement. The most common cell diameters were 23 μm, 21 μm and 24 μm by bright field, and 16 μm, 19 μm and 21 μm by RSOM.

Lastly, we analysed the frequency content of optoacoustic signals generated by single melanocytes to evaluate the need for UWB detection. We compared the signal from a melanoma cell (∼17 μm diameter) at ∼500 μm depth in phantom 2 with a simulated signal from a melanocyte (sphere 20 μm diameter) (Figure 2k,l). Figure 2k shows good agreement between experimental and theoretical waveforms. Figure 2l compares the frequency contents of the simulated and experimental signals. The simulated signal has a primary lobe from ∼0 to 100 MHz, peaking at ∼50 MHz, and a secondary lobe from ∼100 to 180 MHz. The experimental signal has a primary lobe from ∼0 to 120 MHz, peaking at ∼40 MHz, and a secondary lobe from ∼120 to 190 MHz with lower energy due to acoustic absorption and transducer sensitivity. This agreement suggests UWB‐RSOM's unique suitability for this imaging task.

### UWB‐RSOM melanoma imaging and histology

The promising results from synthetic phantom imaging led to an in‐vivo study in humans using 650 nm as the optimal illumination wavelength. Melanocytic lesions, including superficial spreading melanoma, dysplastic nevi and blue nevi (see Appendix S1 for clinical details) on 9 participants, as well as healthy skin from 10 volunteers, were examined. Histological assessments of all lesions were obtained (see Methods) and compared with UWB‐RSOM images. Healthy skin was analysed for signals from non‐melanocytic structures and potential confusion between melanin and haemoglobin signals.

**FIGURE 3:**
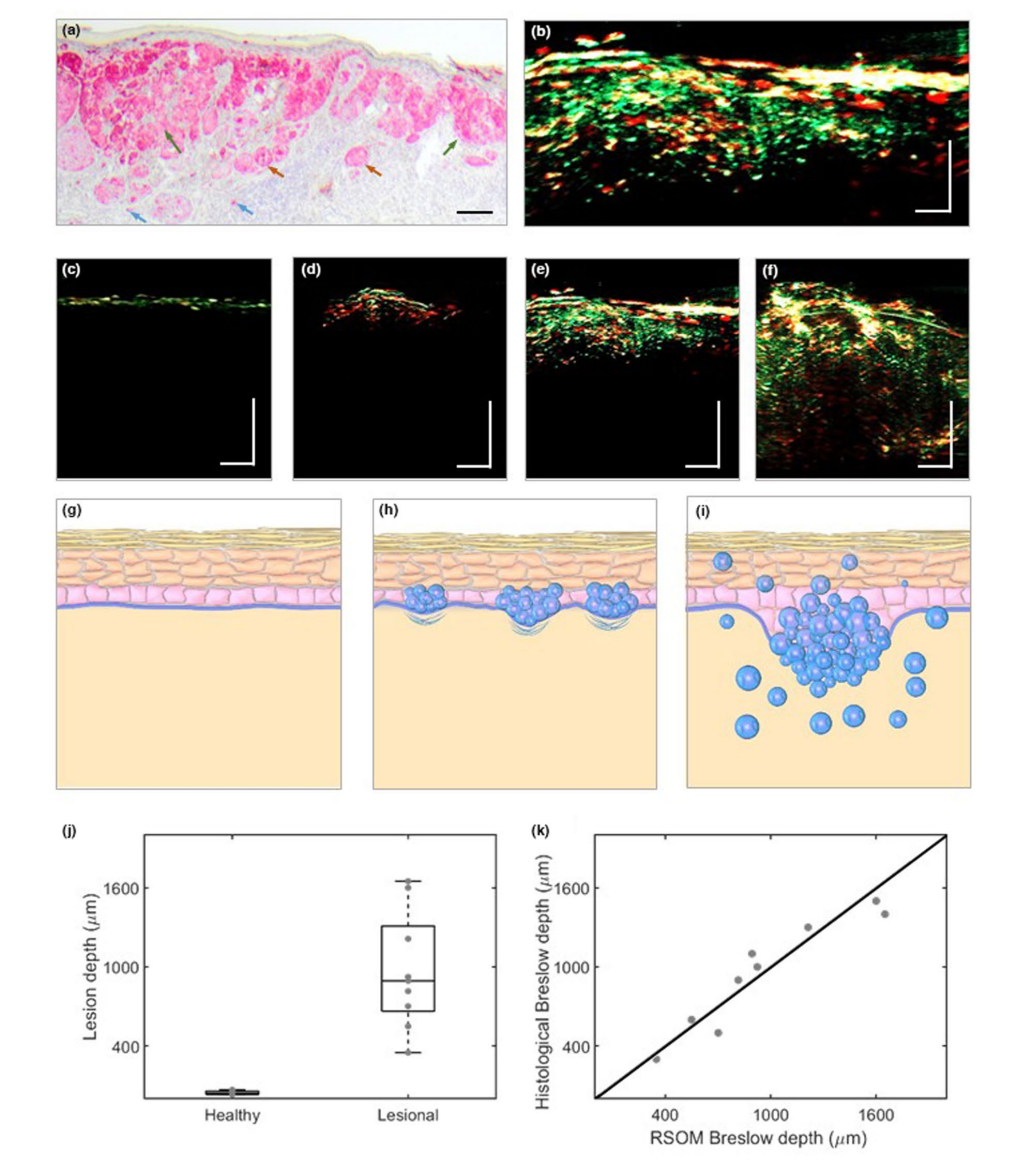
Ultra‐wide bandwidth raster‐scanning optoacoustic mesoscopy (UWB‐RSOM) performance in in vivo melanoma imaging and depth determination. (a) Histological section of a melanoma. Red colour corresponds to melanocytes, the blue arrow indicates individual melanocytes, the orange arrows indicate small nests, the green arrows indicate large nests. (b) UWB‐RSOM cross‐section of the melanoma presented in (a). (c) UWB‐RSOM cross‐sectional image of healthy skin without a melanocytic lesion. (d) UWB‐RSOM cross‐sectional image of a benign dysplastic nevus. (e) UWB‐RSOM cross‐sectional image of a developed melanoma, showing early invasion. (f) UWB‐RSOM cross‐sectional image of a melanoma showing deeper dermal invasion. (g) Scheme of healthy skin showing its different layers. (h) Scheme of a dysplastic nevus, in agreement with the clinical data corresponding to (d). (i) Scheme of a well‐developed melanoma with cancerous melanocytes forming nests in the dermis (large spheres), nodules (group of spheres) or individual scattered cells (small spheres which can be encountered through the whole dermal region). This scheme corresponds to both (e) and (f). (j) UWB‐RSOM‐measured depth of melanocytic structures in healthy skin and skin with lesions. (k) Comparison of the Breslow depths obtained by UWB‐RSOM and histological assessment. Scale bar: 400 μm.

Figure 3a shows a histological section of a superficial spreading melanoma (see Methods), revealing scattered melanoma cells, nests and small nodules. A UWB‐RSOM image of the same lesion displays a similar cellular distribution (Figure 3b). Single cells appear green, whereas larger cells, nests and nodules appear red. UWB‐RSOM's ability to visualize melanocytic lesions is further shown in Figure 3c–f, with images of healthy skin (Figure 3c), a dysplastic nevus (Figure 3d), and two superficial melanomas at early and advanced stages (Figure 3e,f). On healthy skin, nevus melanocytes are at the basal layer of the epidermis (Figure 3g) and are visible by UWB‐RSOM (Figure 3c). In a nevus, melanocyte nests are at the epidermis and/or dermal layers. The typical asymmetrical melanocyte distribution (Figure 3h) is captured by UWB‐RSOM (Figure 3d). In superficially spreading melanoma, some cells can be found in the upper dermis individually or in small nests (Figure 3h). These cells are visible in UWB‐RSOM images as green (single melanocytes) and red dots (larger melanocytes and nests) close to the surface (max depth 0.9 mm) (Figure 3e). At advanced stages (Figure 3i), melanoma cells and nests can be found in the lower dermis, shown in the UWB‐RSOM image (Figure 3f), with single cells (green), nests and small nodules (red) at a max depth of 1.4 mm.

Figure 3j compares the melanin layer thickness in healthy skin (*n* = 10) to the melanocytic penetration depth in lesions, as measured by UWB‐RSOM. The images confirmed that UWB‐RSOM did not visualize the microvasculature at 650 nm due to high CNR from melanin. A visible wavelength is necessary for micro‐vasculature contrast. Comparison of the Breslow thickness determined by UWB‐RSOM and histological assessment (*n* = 9, see Methods and Appendix S1 for details) showed excellent correlation (Figure 3k; *R* ^2^ = 0.886, *p* = 0.0002).

## DISCUSSION

We found that UWB‐RSOM is capable of non‐invasively visualizing single melanoma cells in vivo and determining Breslow thicknesses of melanocytic lesions with unparalleled detail and accuracy. This performance is due to the ultra‐wideband capabilities of the UWB‐RSOM transducers and optimized excitation light wavelength (620–690 nm). The correlation between RSOM and histological Breslow thicknesses was *R* ^2^ = 0.886, likely due to changes in tissue during histological processing.

Our simulations and experimental results show that the ultra‐wideband properties of UWB‐RSOM transducers are essential for detecting the frequency components of optoacoustic signals required to image single melanocytes (frequencies beyond 100 MHz). Previous optoacoustic methods, which used transducers with narrow spectral bands around short central frequencies (21 MHz, 40 MHz, 7.5 MHz), failed to meet AJCC guidelines.

We identified numerically and experimentally the optimal wavelengths for imaging melanocytes as 620–690 nm, and 650 nm was confirmed to be effective, visualizing melanoma cells without haemoglobin signal contamination. In vivo images at 650 nm lacked the micro‐vasculature patterns seen in the visible region UWB‐RSOM images. At 650 nm, RSOM can penetrate through highly absorbing melanocytic lesions, allowing high‐resolution visualization of single cells even in deep skin layers. Moreover, the strong contrast prevents immune cells from altering the depth determination, which is something that may occur in ultrasonography.

UWB‐RSOM is the first non‐invasive method capable of assessing Breslow thickness with resolution and depth that meet general guidelines. Moreover, UWB‐RSOM can visualize the microvascular structure of skin and melanoma^26^, ^27^ and could potentially enhance diagnostics^28^ by simultaneously visualizing melanoma depth and underlying microvascularization using dual‐wavelength RSOM. Moreover, standard dermoscopy could be added to the RSOM system similarly as it is done in (34), for better usability by the practitioners. Nevertheless, the low *n* of the presented study indicates that the results are preliminary. Further work should include a large clinical study including a careful comparison with the performance of ultrasonography. Such study should include tumours with variable depths, a large *n*, and appropriate statistical methods. Also, the possible effect of ulceration in the RSOM images should be investigated.

UWB‐RSOM can address limitations in current melanoma research and health care. Clinically, it could reduce biopsies by identifying melanomas that require large excision margins. Non‐invasive examination benefits clinical research on neo‐adjuvant therapies by allowing the study of tumour response without costly, scarring biopsies that disrupt the tumour microenvironment. UWB‐RSOM could also enhance diagnostics by adding a third dimension to dermatology exams.

## METHODS

### UWB‐RSOM and image reconstruction

For the optoacoustic mesoscopy measurements presented in all figures, we employed a clinical RSOM system described in.^31^ However, the laser was an OPO with a pulse repetition rate of 50 Hz (SpitLight OPO, InnoLas Laser GmbH, Krailling, Germany) to allow imaging in the visible to shortwave infrared range.

For image reconstruction and visualization, we employed the usual filtering strategy in bands (10–40 MHz and from 40 to 120 MHz) and representation in an RGB image 25 (red colour represents large structures, green colour small represents small structures, Figures 2 and 3). The resulting images can be represented in a single‐colour scale (Figure 1) after summing each band.^26^

### OAM of transparent phantoms containing melanocytes

We mixed B16F10 melanocytes at four different concentrations with low melting agar (1.5% agar in distilled water). After solidification on glass‐bottomed petri dishes, we obtained 4 agar slabs (1 cm in height) with the following approximate concentrations of melanocytes: 100, 400, 1600 or 6400 cells/mL (phantoms 1, 2, 3 and 4, respectively, see Appendix S1 and [33,34], for a description of the system).

### Cell concentration calculation and cell size calculation from OAM, RSOM and gold standard

The calculation of the cell concentration in the transparent phantoms using RSOM (Figure 2) was performed by dividing the reconstructed images of phantoms 1, 2 and 3 into 200 μm × 200 μm × 200 μm sub‐sections (the images from phantom 4 were disregarded due to the presence of aggregated cells). For each sub‐section, the number of cells was obtained manually from MIPs. The concentration was calculated from the optoacoustic images in the same fashion.

RSOM reconstructions were used to determine cell sizes (shown in the histogram presented in Figure 2), calculated using the full‐width half‐maximum (FWHM) of profiles drawn in the *Z* direction, for 30 cells randomly selected from phantom 2. Cell sizes were assessed using Countess II (Thermo Fisher Scientific) with automatic settings for illumination and lighting.

### Dermis‐simulating phantom for RSOM and spectrum calculation

In order to obtain the dermis‐simulating phantom used for the spectral calculations shown in Figure 1, we mixed B16F10 melanocytes at an approximate final concentration of 100 cells/μL with low melting agar (1.5% agar in distilled water) together with 10% sheep blood (Sigma‐Aldrich) and 10% intralipid (Braun). After solidification on glass‐bottomed petri dishes, we obtained an agar slab 0.5 cm in height containing well‐separated single B16F10 melanocytes.

Spectral intensities shown in Figure 2k were calculated by summing all the pixel reconstructed values from the regions selected containing a melanocyte shown in Figure 2b–e. The region was drawn in a sagittal MIP.

### Patients and histological Breslow depth calculation

All lesions were diagnosed by professional dermatologists and pathologists based on histological images. The RSOM Breslow depth calculation was performed manually by measuring the distance between the skin surface and the deepest melanocyte (see Figure S2).

We imaged 7 superficially spreading melanomas, 1 benign dysplastic nevus and 1 blue nevus. Those measurements were approved by the Ethics Committee of Klinikum Rechts der Isar der Technischen Universität München, Munich, Germany. The patients in this manuscript have given written informed consent to publication of their case details before starting the measurement. The average age of the patient population was 56.2 ± 12.5 years.

The accurate placement of the scanning head to select the area with the melanocytic lesion for imaging was performed by dermatologists. Diagnoses were determined by dermato‐pathologists via histopathological analysis with H&E and melanin‐specific staining. Histological measurements of lesion depths were performed according to Breslow depth definition, that is, from the stratum granulosum to the deepest point of the nevus, for example, to the basal point of a melanocyte nest. The depth of the lesion was measured by RSOM accordingly from the epidermis to the deepest melanocyte signal. Data sets with low quality, for example, due to motion artefacts, were excluded. For the dysplastic nevus shown in Figure 3d, histological depth was not available.

## AUTHOR CONTRIBUTIONS

**Conceptualization:** Juan Aguirre, Christine Gasteiger. **Data Curation:** Christine Gasteiger, Juan Aguirre. **Formal Analysis:** Juan Aguirre. **Investigation:** Juan Aguirre, Christine Gasteiger, Markus Seeger, Benedikt Hindelang, Andrei Bereznhoi, Ina Weidenfeld, Susanne Annette Steimle‐Grauer. **Software:** Andrei Bereznhoi, Markus Seeger, Juan Aguirre. **Methodology:** Juan Aguirre, Christine Gasteiger, Vasilis Ntziachristos. **Writing—original draft:** Juan Aguirre, Christine Gasteiger. **Writing—review and editing:** Vasilis Ntziachristos, Juan Aguirre, Christian Poch, Tilo Biedermann, Ulf Darsow. **Supervision:** Juan Aguirre, Vasilis Ntziachristos, Tilo Bierdermann, Ulf Darsow. **Visualization:** Juan Aguirre, Markus Seeger. **Resources:** Vasilis Ntziachristos, Andre C. Stiel, Tilo Bierdermann. **Project administration:** Vasilis Ntziachristos, Andre C. Stiel, Tilo Bierdermann.

## FUNDING INFORMATION

This project has received funding from the European Union's Horizon 2020 research and innovation programme under grant agreement nos. 871763 (WINTHER) and 862811 (RSENSE) and under Horizon Europe under no. 101046667 (SWOPT) and by the German Federal Ministry of Education and Research (BMBF) project TEKIOS (grant number 03VP102070). Dr. Aguirre would like to thank support from the Madrid Talento grant 2020‐T1/TIC‐20661TEC.

## CONFLICT OF INTEREST STATEMENT

V.N. is a founder and equity owner of Maurus OY, sThesis GmbH, iThera Medical GmbH, Spear UG and I3 Inc. J.A. and V.N. are inventors on a patent (EP2946721 A1). T.B. received funding from AbbVie, Alk‐Abello, Almirall, Boehringer‐Ingelheim, Celgene‐BMS, GSK, Leo Pharma, Lilly, Novartis, Sanofi‐Genzyme, Regeneron and Viatris. The other authors declare no conflict of interest.

## ETHICAL APPROVAL

The study was approved by the Ethics Committee of Klinikum Rechts der Isar der Technischen Universität München, Munich, Germany.

## ETHICS STATEMENT

The patients in this manuscript have given written informed consent to publication of their case details before starting the measurement.

## Supporting information


Appendix S1.

Video S1.

Video S2.

Video S3.

Video S4.

Video S5.

Video S6.

Video S7.

Video S8.

## Data Availability

The data that support the findings of this study are available from the corresponding author upon reasonable request.
