# Supplementary material for: Non‐invasive characterization of melanoma depth at single‐cell resolution
Source: J Eur Acad Dermatol Venereol. 2025 Jul 3;39(11):1945–54. doi: 10.1111/jdv.20811 (PMC12553128; doi:10.1111/jdv.20811)
Supplement: Supplementary file 1 — Appendix S1. [file JDV-39-1945-s002.docx]

***Non-invasive characterization of melanoma depth at single-cell resolution***

***Supplementary data***

Juan Aguirre, Christine Gasteiger, Benedikt Hindelang, Markus Seeger, Andrei Bereznhoi, Ina Weidenfeld, Ulf Darsow, Andre C. Stiel, Susanne Annette Steimle-Grauer, Andre C. Stiel, Christian Posch, Tilo Biedermann, Vasilis Ntziachristos

**Supplementary Note 1: Optoacoustic microscopy (OAM) imaging system**

In Suppl. Fig. 1, we depict a section of the optoacoustic (OA) microscope relative to the objective of the microscope, the petri dish containing the transparent phantom with the mixture of melanoma cells and the ultrasound transducer.

**
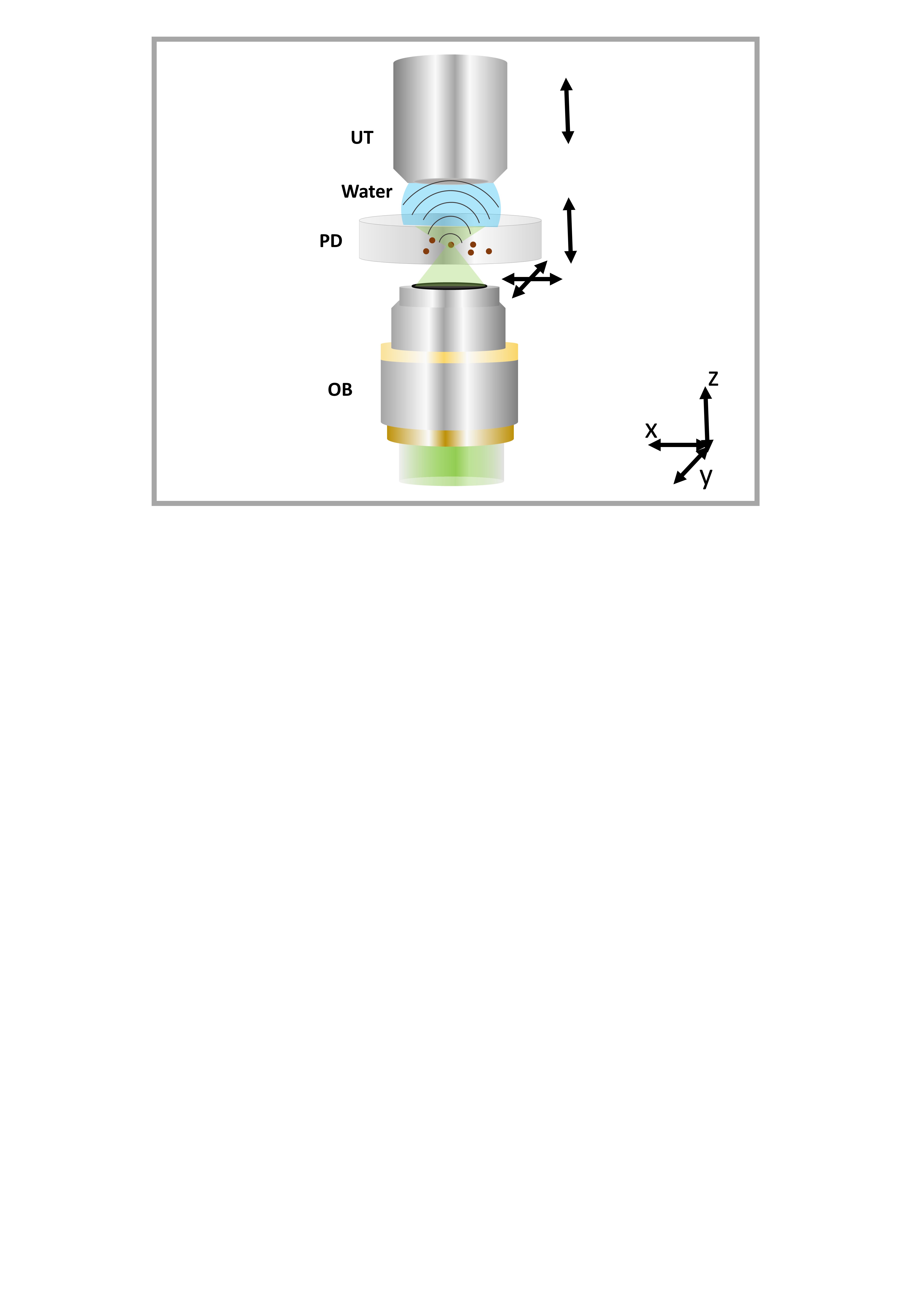
**

**Supplementary Figure 1**: Depiction of the arrangement of the imaging framework and schematic depiction of the optoacoustic (OA) system. The excitation light beam (represented in green) is directed into the inverted microscope objective (OB) after being conditioned by a set of lenses (see Methods). A galvo scanning system allows the scanning of the focal point in the x-y plane. A petri dish with a mixture of agar and melanocytes (PD) is coupled acoustically to the ultrasound transducer (UT) by a water droplet. Both the petri dish and the transducer can move in the z-direction using a common stage that allows the scanning of the focal point from different heights.

**Supplementary Note 2: Clinical information from imaged melanocytic lesions**

In Suppl. Table 1, we show the clinical diagnosis and age of each patient enrolled in the study.

**Supplementary Table** **1**: Clinical details of all patients imaged in this study

| Patient number | Histological diagnosis | Age (years) |
| --- | --- | --- |
| 1 | Superficially spreading melanoma | 52 |
| 2 | Benign dysplastic nevus  (histological depth not available) | 59 |
| 3 | Superficially spreading melanoma | 58 |
| 4 | Superficially spreading melanoma | 74 |
| 5 | Blue nevus | 70 |
| 6 | Superficially spreading melanoma | 35 |
| 7 | Superficially spreading melanoma | 43 |
| 8 | Superficially spreading melanoma | 51 |
| 9 | Superficially spreading melanoma | 64 |

In Suppl. Fig. 2, we show the appearance of the imaged blue nevus under a standard dermatoscope and under raster-scan optoacoustic mesoscopy (RSOM). The method for calculating the Breslow depth of the blue nevus consisted of measuring the distance from the skin surface to the deepest cell in the cross-sectional maximum intensity projection (MIP). The same procedure was performed for the all melanoma images used in this study.


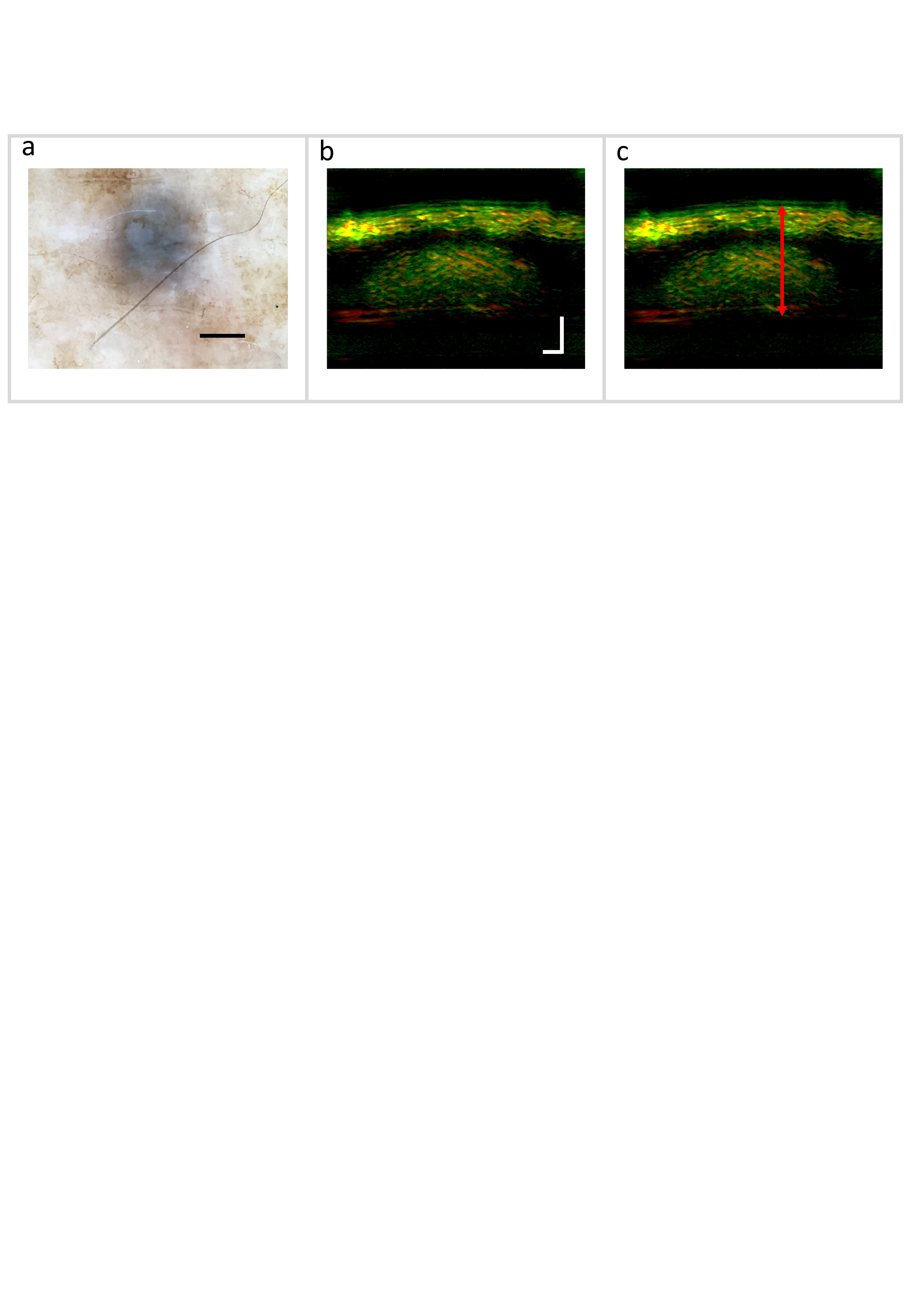


**Supplementary Figure 2:** Representative image of a blue nevus, showcasing the method used to obtain the Breslow depth. **a)** Dermatoscopic image of a blue nevus. Scale bar: 1mm **b)** Raster-scan optoacoustic mesoscopy (RSOM) cross-sectional maximum intensity projection of the blue nevus. Scale bars: 300 μm. **c)** Calculation of the Breslow depth. The Breslow depth is calculated as the distance from the skin surface to the deepest cell, as depicted by the red arrow.
